# Association between depressive symptoms and all-cause mortality in Chilean adult population: prospective results from two national health surveys

**DOI:** 10.1007/s00127-023-02534-9

**Published:** 2023-07-20

**Authors:** Eliazar Luna, Hynek Pikhart, Anne Peasey

**Affiliations:** 1 Department of Clinical, Educational and Health Psychology, University College London, 1-19 Torrington Place, London, UK; 2 Department of Epidemiology and Public Health, University College London, 1-19 Torrington place, London, UK

**Keywords:** Depression, Mortality, Mental health, Latin America, Survival

## Abstract

**Purpose:**

Depression is a prevalent disorder with effects beyond mental health. A positive association with mortality has been mostly reported, however, evidence comes from a few high-income countries. This study aims to assess the association between depressive symptoms and all-cause mortality in the Chilean population and assess a potential secular effect in this association.

**Methods:**

This prospective study used data from the Chilean National Health Survey (CNHS). Data from 3151 and 3749 participants from the 2003 and 2010 CNHS, respectively, were linked to mortality register data. Cox survival analysis was performed. The main exposure was depressive symptoms, measured with CIDI-SF (cut-off ≥ 5), and the outcome all-cause mortality. The study period was limited to 8.5 years to allow for the same length of follow-up.

**Results:**

10% and 8.5% of participants from the 2003 and 2010 cohort died during the follow-up. Adjusting for age and sex, those with depressive symptoms had 1.58 (95% CI 1.18–2.13) and 1.65 (95% CI 1.14–2.12) times the risk to die than those without symptoms in the 2003 and 2010 cohort, respectively. In models adjusted for demographic, socioeconomic, behavioural variables and comorbidities, participants with depressive symptoms had 1.42 (95% CI 1.05–1.92) and 1.46 (95% CI 1.07–− 1.99) times the risk to die compared to those without symptoms in the 2003 and 2010 cohort, respectively.

**Conclusion:**

Chilean adults with depressive symptoms are at higher risk of all-cause mortality compared to those without symptoms. The effect size was similar regardless of the economic development of the country.

**Supplementary Information:**

The online version contains supplementary material available at 10.1007/s00127-023-02534-9.

## Background

Depression is a common mental health condition with a range of effects on quality of life, disability, healthcare costs, and mortality, among others [1]. This paper examines the association between depression and mortality. Numerous studies have suggested those with depression tend to have a shorter lifespan than those without depression [2]. Despite the relatively high number of studies assessing the association between depression and mortality, there is a large gap as evidence mostly comes from a few high-income Western countries [2–7]. Reviews focusing on populations from low-and-middle-income countries (LMIC) are limited to about 10 studies [8], while reviews encompassing studies mostly from high-income Western countries include over 290 studies [2]. Moreover, there are methodological limitations in studies reported from countries with a similar income or from the same region as Chile [8]. Chile is a Latin American country that has undergone a substantial economic transition, from a LMIC in the early 2000s to an upper-middle-income country in the first decade of this century and ultimately to a high-income country in 2013 [9]. Due to this economic transition, Chile offers a unique opportunity to assess the association between depression and mortality.

The estimated effect size of the association between depression and mortality varies widely between studies. Reports of positive associations range between relative risk (RR) of 1.1 to 3.6 [5], with most studies reporting a positive association A meta-analysis pooling 238 studies reported an estimated RR of 1.64 (95% CI 1.56–1.76) [2], while a meta-analysis focusing exclusively on studies assessing the effect of clinically defined major depression reported an RR of 1.92 (95% CI 1.65–2.23) [3], suggesting severity may influence the association with all-cause mortality. This is because studies measuring depression through screening instruments tend to also capture less severe forms of depression or subclinical depressive symptoms [3].

Emerging evidence suggests that the widely reported positive association between depression and mortality is heavily determined by the quality of the evidence. This is based on elements such as number of deaths, influence of small studies, excess of significance and heterogeneity. In the umbrella review by Machado and colleagues, this positive association was found to be highly influenced by reviews of lower quality [5]. Estimates considering the quality of evidence consistently differed, with evidence showing smaller effect sizes in high-quality studies than in studies with low quality. Several reviews have outlined the indirect association between quality of evidence and size of the effect [2, 4, 5, 8], with pooled estimates from high-quality studies being up to 36% smaller than pooled estimates from low-quality studies [2].

Most authors have identified inconsistencies in the association between depression and mortality [2, 4, 6, 8, 10] with several factors contributing to this, such as measurement of exposure, sample size, type of population, follow-up length, publication bias, outliers and adjustments [5, 11]. For type of population, there is evidence of a smaller effect for community samples compared to population with a specific disease [2]. As for follow-up, studies with a longer follow-up tend to yield smaller effect sizes than studies with a short follow-up [5]. There is also evidence of smaller effect sizes when removing outliers [2, 8]. A meta-analysis reported an attenuation of the estimated RR from 1.64 (95% CI 1.56–1.72) to 1.58 (95% CI 1.51–1.65) after removing outliers [2]. Most importantly, lack of adjustment for health conditions and health behaviours has been consistently identified as a relevant limitation [5].

An examination of studies from Latin America shows that none of them are nationally representative, most studies have focused on older populations [12–16] or in-patient samples [17–21], and none of them were conducted in Chile. The vast majority of studies have sample sizes of less than 500 people [13, 17–22]. A Brazilian study of older adults with 10 years of follow-up is among the few without some of these limitations [14]. Here, those with depressive symptomatology had 1.24 (95% CI 1.00–1.55) times the risk of dying during the follow-up compared to those without symptomatology after adjusting for demographic, socioeconomic, functional limitations, cognitive features, lifestyle factors and chronic diseases. Other Latin-American research has reported both positive and no association between depression and mortality [14, 15, 19, 20]. Studies reporting no association were from in-patient samples [17, 18, 21, 22] rather than community samples.

Most evidence with mortality as outcome focuses either on physical health or severe mental illnesses as exposure [23, 24]. Moreover, there are important limitations to the evidence at the global and, more importantly, at the Latin American level. This highlights the relevance of conducting a study on the topic in Chile to address these common methodological shortcomings. Based on the hypothesis of a higher risk of mortality of those with depressive symptoms compared to those without, the purpose of this paper is to extend the existing literature base and contribute to addressing the paucity of evidence from Latin America, using high-quality data from two Chilean nationally representative samples, with a specific measure of depressive symptoms, using a time-to-event analysis of all-cause mortality, and adjusting for a range of demographic, socioeconomic, behavioural confounders and comorbidities. Moreover, the use of two different surveys from the same population enables us to address a potential secular effect on the association between depressive symptoms and all-cause mortality in the Chilean population.

## Methods

### Study population

The study sample came from the 2003 and 2010 Chilean National Health Surveys (CNHS). These are random, geographically stratified, multi-stage cross-sectional surveys representative of Chilean adult population. The detailed study protocol of each CNHS is described elsewhere [25, 26]. After exclusions due to missing exposure, outcome and covariates, the original survey samples—*n* = 3,619 and *n* = 5,293—, were reduced to *n* = 3,151 and* n* = 3,749 for the 2003 and 2010 CNHS analytical sample, respectively. This study used a complete case approach, meaning that all descriptive and inferential analyses are based on sample size of the analytical samples.

### Exposure: depressive symptoms

Depressive symptoms were measured by trained interviewers at baseline using the Latin American version of the Composite International Disease Instrument Short Form (CIDI-SF) [27, 28], a screening instrument with a score ranging from 0 to 7, with a traditional cut-off indicating probable cases of depression of 5 [29]. Therefore, a binary exposure variable was created using this cut-off to indicate those with depressive symptoms.

### Outcome: time to death

Mortality data were provided by the Chilean Ministry of Health, who linked the participant’s CNHS identifier with administrative mortality data. This linkage data contained a variable for mortality, date and cause of death according to ICD-10 classification [30]. The time-to-event variable was estimated using the number of days from the interview date to the endpoint for the study (either the date of death or the end of follow-up) and follow-up was censored to 8.5 years in both CNHS, the longest possible follow-up length of participants of the 2010 CNHS. This is essential to assess a potential secular effect in the association between depressive symptoms and all-cause mortality, as there is evidence of a negative association between length of follow-up and size of the effect [5]. The same follow-up length allows comparable results between cohorts. As participants were followed over time, the surveys will be referred to from now on as 2003 and 2010 cohorts.

### Covariates

The demographic covariates were age in years, sex and marital status (living with partner, widowed/divorced, no partner). Socioeconomic status (SES) covariates were years of education (< 8 years, 8–12 years, 13 + years) and working status (employed/student, homemaker, retired, unemployed). Lifestyle variables included were physical activity (3 + times weekly, 1–2 times weekly, < 4 times per month, no physical activity) and smoking (smoker and non-smoker). Statistical models were also adjusted for the chronic diseases of type II diabetes and high-blood pressure (HBP), ascertained through physiological measurements and the use of prescribed medicine for these conditions. The reference groups for the statistical models were being female, living with a partner, 13 + years of education, employed/student, engaging in physical activity 3 + times weekly, non-smoker, and without HBP, diabetes and depressive symptoms.

### Statistical analyses

#### Descriptive analysis

The sample characterization was displayed by cohort. Kaplan–Meier plots representing the association between each category of exposures and mortality over time were analyzed. For the mortality data, the number and proportion of people who died and the survival function by cohort were inspected. Lastly, the survival function by cohort was examined. This function allows examining the probability of survival for participants over time.

#### Multivariable regression analysis

To assess a potential association between depressive symptoms and all-cause mortality, four models were fitted using Cox proportional hazard models [31], with participants with CIDI-SF < 5 as the reference group. The aim of the study and the nature of the outcome—time-to-event—make Cox survival analysis an appropriate method to use [32]. Firstly, a model adjusted by age and sex (model 1) was built, then it adjusted for marital status and SES variables (model 2), after that, health behavioural variables were added (model 3) and, lastly, adjustments for chronic diseases were made (model 4). Interactions between the exposure, age and gender were examined but no evidence of such interactions was found.

One of the key aspects of Cox proportional hazard model is the proportional hazard assumption. This was assessed by examining log–log plots, including a time interaction in the model, performing the non-zero slope of the Schoenfeld residuals test [33] and graphically examining scaled Schoenfeld residuals. The assumption was tested in the fully adjusted model.

#### Sensitivity analyses

Sensitivity analyses were carried out to assess potential biases in the study. To assess representativeness of the analytical sample, a comparison with excluded participants by survey was made using chi-square or chi-square for trend. To examine a potential reverse causality, we excluded those who died in the first 6 months. These models were adjusted only by age and sex to avoid statistical power issues. All analyses were done using RStudio version 1.4.

## Results

### Sample characterization

For the 2003 (*n* = 3,151) and 2010 (*n* = 3,749) cohorts, there were 25,505 and 30,578 person-years of observation, respectively. About 16% of the sample in each cohort had depressive symptoms. For illustrative purposes, age was categorized into three groups: 18–44, 44–65 and 65 + years old**.** The age range of participants was 18 to 100 years old at baseline. There were some slight differences in the characteristics of the analytical samples for the two cohorts (Table 1). The main difference related to SES: the 2010 cohort had a lower proportion of unemployed people and a higher proportion of people with 13 + years of education compared to the 2003 cohort. The bivariate association between the different variables and mortality was assessed through Kaplan–Meier plots. Supplementary Fig. 1 shows the Kaplan–Meier plots for each variable. Results of these plots suggest that groups with poorer health or lower SES tend to have shorter survival over time.

**Table 1:** Sample characterization of the analytical sample by cohort

Variable	Category	2003 Cohort *n* = 3,151			2010 Cohort *n* = 3,749		
		*n* (%)	Person-years	Deaths per 1,000 person-years	*n* (%)	Person-years	Deaths per 1,000 person-years
Depressive symptoms (CIDI-SF ≥ 5)	Non depressed	2,638 (83.7)	21,401	12.2	3,136 (83.7)	25,605	10.4
	Depressed	513 (16.3)	4,104	13.4	613 (16.4)	4,973	10.5
Sex	Females	1,721 (54.6)	13,988	11.4	2,227 (59.4)	18,219	9.5
	Males	1,430 (45.4)	11,517	13.5	1,522 (40.6)	12,359	11.7
Age groups (years)	18–44	1,344 (42.7)	11,399	0.8	1,742 (46.5)	14,742	0.9
	45–64	1,024 (32.5)	8,517	5.5	1,282 (34.2)	10,607	6.8
	65 +	783 (24.9)	5,590	46.3	725 (19.3)	5,230	44.6
Marital status	With partner	1,866 (59.2)	15,273	9.7	2,203 (58.8)	18,124	8.2
	Widowed/Divorced	529 (16.8)	3,929	34.4	712 (19.0)	5,498	24.4
	No Partner	756 (24.0)	6,303	4.9	834 (22.2)	6,956	5.2
Years of education	13 +	389 (12.4)	3,279	2.4	738 (19.7)	6,186	3.6
	8–12	1,144 (36.3)	9,552	5.1	2,012 (53.7)	16,675	5.9
	Less than 8	1,618 (51.4)	12,675	20.4	999 (26.7)	7,717	25.7
Working status	Employed + Student	1,396 (44.3)	11,744	2.7	2,046 (54.6)	17,151	3.7
	Homemaker	935 (29.7)	7,638	10.9	912 (24.3)	7,578	6.1
	Retired	433 (13.7)	3,035	50.4	578 (15.4)	4,196	41.9
	Unemployed	387 (12.3)	3,088	15.2	213 (5.7)	1,653	20.0
Physical activity	3 + times weekly	262 (8.3)	2,208	3.6	274 (7.3)	2,306	2.2
	1–2 times weekly	305 (9.7)	2,546	5.5	280 (7.5)	2,370	0.8
	< 4 times per month	131 (4.2)	1,096	2.7	172 (4.6)	1,438	4.2
	No sport	2,453 (77.9)	19,656	14.8	3,023 (80.6)	24,463	12.5
Smoking	Non-smoker	2,052 (65.1)	16,332	16.6	2,395 (63.9)	19,291	13.5
	Smoker	1,099 (34.9)	9,173	4.8	1,354 (36.1)	11,287	5.1
High-blood pressure (HBP)	No	2,068 (65.6)	17,056	7.3	2,694 (71.9)	22,391	5.3
	Yes	1,083 (34.4)	8,449	22.5	1,055 (28.14)	8,187	24.3
Type II Diabetes	No	2,832 (89.9)	23,176	10.0	3,392 (90.48)	27,851	8.6
	Yes	319 (10.1)	2,329	36.1	357 (9.52)	2,727	28.6

### Mortality characterization

In total, 633 participants (315 and 318 in the 2003 and 2010 cohort, respectively) died during the 8.5-year follow-up. The all-cause data suggested a gradual divergence in mortality between cohorts in absolute terms. The proportion of people who died was very similar after a 2-year follow-up, but this difference increased over time. People from the 2003 cohort had higher mortality—of about 1.5%—compared to the 2010 cohort. (Table 2) Details of the proportion of deaths by covariates can be found in Supplementary Table 1. The highest proportion of deaths for each covariate was for the category of males, being older, being widowed/divorced participants, being retired, having less than 8 years of education, not engaging in physical sport, not smoking, having HBP, type-II diabetes in both 2003 and 2010, while those with depressive symptoms had a higher proportion of deaths compared to those without these symptoms only in 2003. Unlike the Cox models presented in a later section, this examination does not take confounding into account.

**Table 2:** Number and percentage of all-cause mortality by follow-up time and cohort

CNHS	2 years	5 years	8.5 years
2003 (*N* = 3,151)	77 (2.44%)	174 (5.52%)	315 (10.00%)
2010 (*N* = 3,749)	68 (1.81%)	169 (4.51%)	318 (8.48%)

### Survival function examination

**Fig. 1:**
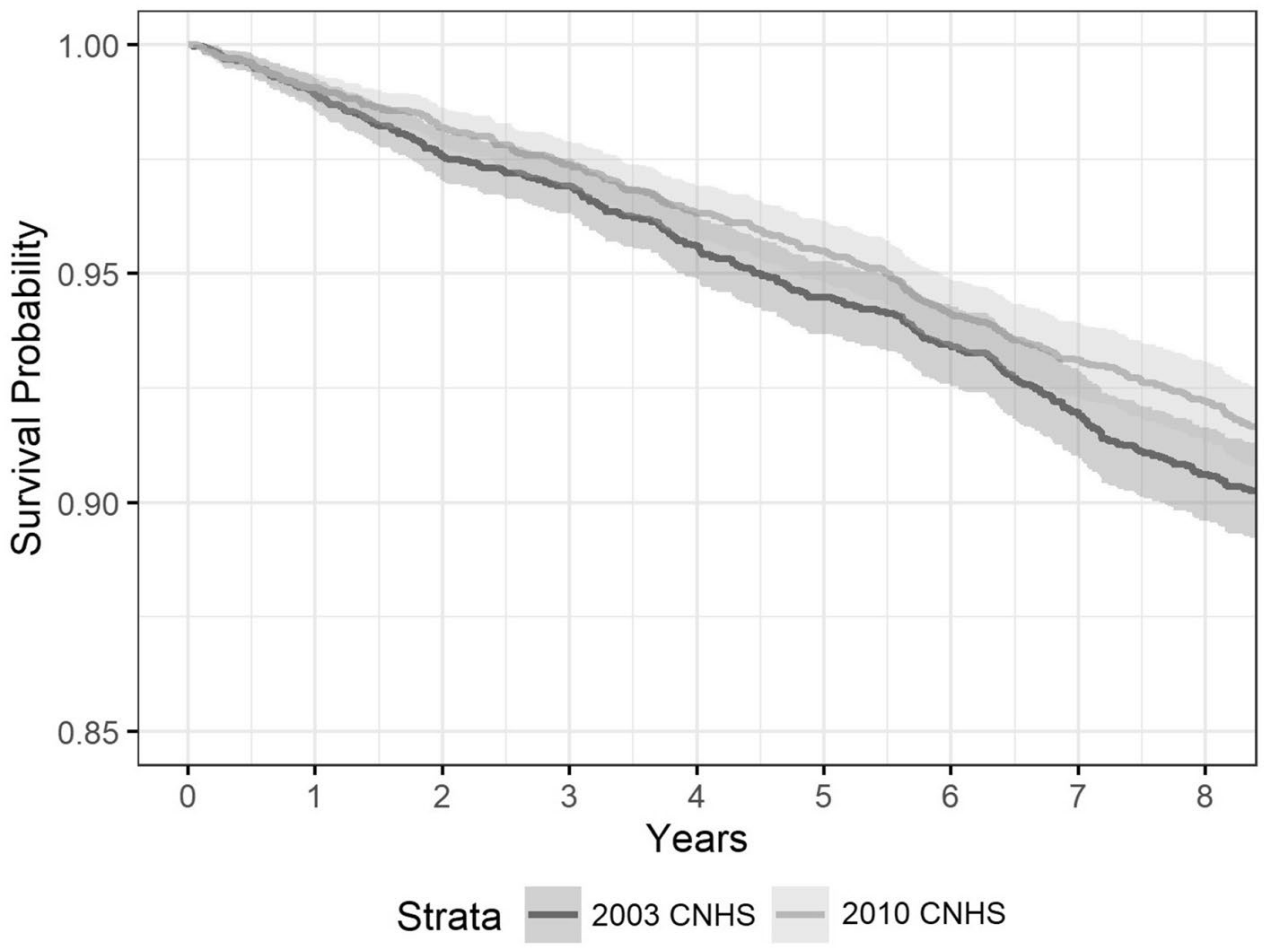
Survival function by CNHS cohort

Mortality by cohort was assessed with the survival function. Figure 1 compares the survival function of the 2003 and 2010 cohort within the 8.5-years follow-up period. The survival function seems to be lower for the 2003 cohort than for the 2010 cohort. After the follow-up period, there was a 90% and a 91.5% of survival probability, respectively. Nevertheless, the overlapping of confidence intervals between cohorts suggests that the survival functions are statistically indistinguishable.

### Cox Models for the association between depressive symptoms and all-cause mortality with an 8.5-year follow-up

Table 3 shows the estimated hazard ratio (HR) for the association between depressive symptoms and all-cause mortality for all models. Model 1, adjusted by age and sex, suggests a positive association between depressive symptoms and all-cause mortality in both cohorts. Those with depressive symptoms had 1.58.

**Table 3:** Association between depressive symptoms and all-cause mortality with an 8.5-year follow-up by cohort

	2003 cohort (*n* = 3,151)		2010 cohort (*n* = 3,749)	
	HR (95% CI)	*P*-value	HR (95% CI)	*P*-value
Model 1	1.58 (1.18 – 2.13)	0.002	1.65 (1.22 – 2.23)	0.001
Model 2	1.50 (1.11 – 2.02)	0.008	1.56 (1.14 – 2.12)	0.005
Model 3	1.49 (1.10 – 2.00)	0.010	1.52 (1.11 – 2.06)	0.008
Model 4	1.42 (1.05 – 1.92)	0.022	1.46 (1.07 – 1.99)	0.017

*Model 1* Model adjusted for sex and age. *Model 2* Model additionally adjusted for area of residence, marital status and the SES variables of education and working status. *Model 3* Model additionally adjusted for the behavioural variables of physical activity and smoking. *Model 4* Model additionally adjusted for the comorbidities of high-blood pressure and diabetes.

(95% CI 1.18–2.13) and 1.65 (95% CI 1.22–2.23) times the risk of mortality compared to those without such symptoms in 2003 and 2010, respectively. Subsequent adjustments for marital status and SES variables (model 2) substantially attenuated the estimated effect size. Those with depressive symptoms had 1.54 (95% CI 1.11–2.02) and 1.56 (95% CI 1.14–2.12) times the risk of mortality compared to those without depressive symptoms in the 2003 and 2010 cohorts, respectively. Further adjustment for physical activity and smoking (model 3) slightly attenuated the effect, as the estimated HR for these models were 1.49 (95% CI 1.10–2.00) and 1.52 (95% CI 1.11–2.06) in 2003 and 2010, respectively. Lastly, adjustments for the presence of HBP and diabetes attenuated the estimated effect (model 4). For the 2003 cohort, those with depressive symptoms had 1.42 (95% CI 1.05–1.92) times the risk to die at any point during the 8.5-year follow-up period than those without depressive symptoms. 2010 participants with depressive symptoms had 1.46 (95% CI 1.07 –1.99) times risk of mortality over the analyzed period compared to those without such symptoms. In the fully adjusted model, the data suggest that the effect of depressive symptoms on mortality was very similar between the older (2003) and newer (2010) cohort. Based on the small difference in the estimated effect, evidence for a secular trend in the association between depressive symptoms and all-cause mortality of Chilean adult population is very limited.

Supplementary Table 2 contains the estimated HR for all covariates in the fully adjusted model for the 2003 and 2010 cohorts. In these models, being older, male, unemployed, retired, not engaging in physical activity, and having diabetes were associated with a higher risk of mortality compared to participants who were younger, female, employed, physically active or did not have diabetes in fully adjusted models. The variables with the largest effect on mortality were age, employment status and sex. 1-year increase in age was associated with 1.09 (95% CI 1.08–1.10) times the risk of dying, unemployed participants had up to 3.35 (95% CI 2.13–5.26) times the risk of mortality compared to employed participants and males had up to 1.61 (95% CI 1.24–2.08) times the risk of mortality compared to females; after adjusting for all other variables. Lastly, there was no evidence of a violation of the proportional hazard assumption of Cox models in the fully adjusted model.

### Sensitivity analyses

The comparison of the sample excluded due to missing data and the analytical sample of the two cohorts showed good agreement between them. There is no evidence that excluded participants had systematically poorer health than the analytical sample. The sensitivity analysis excluding those who died in the first 6-months of the follow-up—to assess reverse causality—supported our findings. Moreover, only one of the excluded participants died from reasons other than health. Details on the estimates of this sensitivity analysis can be found in Supplementary Table 3. The estimated HR for the effect of depressive symptoms on all-cause mortality was somewhat attenuated in this analysis, yet it was robust to the aforementioned exclusion of participants.

## Discussion

The present study is the first to assess the association between depressive symptoms and all-cause mortality in the Chilean population. The mortality risk remained elevated for those with depressive symptoms after adjusting for demographic, SES, behavioural and chronic conditions in the 8.5-year period analysed. There was consistent evidence of an increased risk of all-cause mortality for those with depressive symptoms compared to those without such symptoms in both cohorts, suggesting no secular effect. This study adds to the body of literature suggesting a higher risk of mortality of depressed individuals compared to those without this condition. Moreover, this is the first study conducted in not one but two nationally representative samples from a Latin American country and, as far as we know, the first one to assess the association between depressive symptoms and mortality in a country that transitioned from a LMIC to a HIC. This study suggests that the elevated risk of mortality is similar regardless of the economic development stage in Chile.

The results from our work agree with the hypothesis of a higher risk of all-cause mortality for depressed individuals compared to those without. This hypothesis has been previously tested with mostly positive results, however, these studies had the limitations previously discussed in the background section [2, 5]. Our fully adjusted estimates are lower than Cuijpers’ review estimate [2] but the latter was attenuated after removing outliers and adjusting for publication bias (RR 1.58 95% CI 1.51–1.65). Compared to literature from LMIC, the estimates from our work also tended to be smaller. After adjusting for outliers and publication bias, the review of the topic from LMIC reported a pooled RR of 1.60 (95% CI 1.37–1.86). Nevertheless, when only studies of high-quality are considered, the estimate decreased to 1.48 (95% CI 1.32–1.67).

Although pooled estimates from the literature tend to be larger in size than that estimated from our work, when critically assessed and adjusted for relevant factors, there is a much better agreement with our results. Most estimates from the literature are only adjusted by age and sex [4] and do not address the limitations described in the background, such as measurement of exposure, sample size, type of population, follow-up length, publication bias, outliers and adjustment for confounders [5, 11]. When only age and sex adjustments are considered, model 1, our estimate shows good agreement with the estimate from Cuijpers’ review after adjustments for publication bias and outliers. The estimates of the association between depression and mortality in the literature are relatively similar when elements such as quality of studies and variables adjusted for are considered [2, 8]. This strengthens the generalisability of our results and suggests that, rather than specific mechanisms that influence all-cause mortality, more generic mechanisms drive the association between depression and mortality [2].

The association between depression and mortality is hypothesized to be explained by two non-mutually exclusive mechanisms: biological dysregulation and unhealthy behaviours [3]. The former suggests that depression causes changes at the biological level, such as alterations in inflammatory responses [34], in the hypothalamic–pituitary–adrenal (HPA) axis [35, 36], cortisol levels [37] and noradrenaline levels [38] and these biological changes increase the risk of mortality. The latter encompasses unhealthy behaviours ranging from lack of physical activity [39, 40], smoking [41, 42] to alcohol and drug abuse [43, 44]. In turn, these behaviours associated with depression, increase the risk of mortality. In this paper, the effect of certain unhealthy behaviours—smoking and physical activity—and chronic diseases strongly associated with unhealthy behaviours—diabetes and HBP—was accounted for. Thus, the estimated effect of depression on all-cause mortality in our model was independent of these factors. However, if unhealthy behaviours, such as lack of exercise, mediate the association between depression and mortality, adjusting for chronic conditions also associated with those unhealthy behaviours could potentially lead to overadjustment of our results [45]. The comparison of models with and without adjustments for unhealthy behaviours shows an inconsequential attenuation of the estimated effect, suggesting that, after accounting for depressive symptoms, demographic and SES variables, these unhealthy behaviours have little effect on all-cause mortality in our data. On the other hand, adjustment for chronic diseases strongly linked to unhealthy behaviours showed a small attenuation of the estimated effect between depression and mortality. Therefore, a potential overadjustment due to consideration of potential mediators in the model was considered to be negligible.

Despite some consistency in the positive association between depression and mortality reported in the literature [4, 46], Machado et al. emphasized that quality of studies could influence the size of this association [5]. This is true to some extent, as evidence from Machado’s and other reviews suggest a larger effect of studies of low-quality compared to high-quality studies. Nevertheless, in sensitivity analysis adjusted for comorbidities in community samples, the evidence from the umbrella review [5] still provides evidence for a positive association between depression and mortality, with a reported effect size similar to our work (RR 1.38, 95% CI 1.29–1.47). It has also been posited that the increased mortality risk among depressed individuals is caused by other physical disorders that in turn cause depression [7]. However, both our estimate for the effect of depressive symptoms on mortality was robust to adjustment for chronic diseases and our sensitivity analysis excluding those who died in the first six months to address reverse causality support the robustness of the results that depression increases mortality risk.

### Strengths

There were several strengths in this study. This is the first Latin American study on the topic to be conducted on two nationally representative samples, enhancing the generalisability of its results at the national level. There is also potential for replicability of this study in other countries from Latin America. Mexico, for example, has good-quality data from surveys similar to the ones used in these analyses [47] and can assess the association between depressive symptoms and all-cause mortality in the Mexican population. Moreover, both cohorts had a large sample size and there were enough deaths to ensure an appropriate power for this study and the use of two cohorts also allowed examination of potential secular trends. The main exposure showed a remarkably stable effect on mortality. By restricting the follow-up up to 8.5 years, reasonable comparisons between cohorts could be made, as there is evidence of changes in the estimated effect size according to the length of the follow-up period [2]. Attrition has been consistently identified as a limitation in prospective studies [48]. As the mortality data of CNHS’ participants were linked using administrative data and not by contacting all participants at follow-up, attrition was not a limitation. Furthermore, this study used reliable data. Diabetes and HBP were ascertained through physiological measurements made by a nurse instead of self-reporting. One of the most consistent limitations highlighted in the literature was the lack of adjustment for relevant confounders. In this paper, a wide variety of confounders was included from different domains: demographic, socioeconomic, lifestyle variables and chronic conditions. The attenuation between model 1 and the fully adjusted model suggested that these adjustments—especially the SES variables—were relevant to assess the association between depression and mortality.

### Limitations

There were, however, some limitations to this study. Some pertinent variables were unavailable or had high level of missingness and could not be used in the analysis, such as diet, cardiovascular diseases, Alzheimer’s disease, chronic obstructive pulmonary disease, and other mental health conditions [2, 5]. Therefore, residual confounding cannot be discarded. Also, although the survival probability of each cohort seemed to diverge over time, the overlapping of the estimated confidence intervals at the end of the examined period does not allow us to say that they are different. The instrument for measuring our exposure was not a structured psychiatric interview, but rather a screening instrument. This has the potential limitation of misclassification of subjects compared to the gold standard. However, there is evidence that the used instrument shows a high level of agreement with structured psychiatric interviews [29]. The measurement of depressive symptoms at baseline could reflect either an acute state at the moment of interview or the presence of a more chronic condition. The trajectories of depressive symptoms cannot be ascertained by our design, consequently, it was not possible to assess how much of the estimated effect of depressive symptoms on mortality is due to an acute or repeated depressive episodes. Public health policies could focus on either prevention—if the mere presence of depression increases risk of mortality—or management of depressive episodes if repeated episodes are more relevant for mortality. Overadjustment based on including potential mediators in the association between depressive symptoms and mortality could underestimate the true association. However, as previously discussed, the potential overadjustment due to the inclusion of potential mediators of unhealthy behaviours and chronic conditions in the models 3 and 4, respectively, was judged to be negligible based on the small attenuation of the estimates in our analyses. The potential bias due to the incompleteness of mortality records was addressed by examining the quality of the data in terms of cause and number of deaths. Overall, there was a relatively constant number of deaths over time and a similar proportion in causes of death by cohort. The small differences in these aspects were considered irrelevant. Lastly, there is some evidence of a difference in the effect of depression on mortality depending on the cause of mortality and gender [46]. Our analysis did not consider these aspects solely based on a lack of power to detect significant differences for a more specific outcome, such as cardiovascular mortality, or by gender.

## Conclusion

This study showed a similar effect of depressive symptoms on mortality between the 2003 and 2010 cohorts of Chilean population over an 8.5-year period. The effect size was similar in size despite the economic development of Chile and similar in size to what has been reported in other high-income Western countries. Future research in this population could focus on assessing the effect of trajectories of depressive symptoms on mortality through a design that considers data collection at more points over time.

### Supplementary Information

Below is the link to the electronic supplementary material.

Supplementary file1 (DOCX 412 KB)

## Data Availability

CNHS’ data are publicly available on the website of the Chilean Department of Epidemiology, http://epi.minsal.cl/bases-de-datos/. Data on mortality is not publicly available but it can be requested to the Ministry of Health through the Transparency Law platform.
