# Supplementary material for: Association between depressive symptoms and all-cause mortality in Chilean adult population: prospective results from two national health surveys
Source: Soc Psychiatry Psychiatr Epidemiol. 2023 Jul 20;59(6):1003–12. doi: 10.1007/s00127-023-02534-9 (PMC11116228; doi:10.1007/s00127-023-02534-9)
Supplement: Supplementary file 1 — Supplementary file1 (DOCX 412 KB) [file 127_2023_2534_MOESM1_ESM.docx]

Association between depressive symptoms and all-cause mortality in Chilean adult population: prospective results from the National Health Survey.

**Journal of Social psychiatry and psychiatric epidemiology**

**Authors:** Eliazar Luna^1^, Hynek Pikhart^1^, Anne Peasey^1^

^1^Department of Epidemiology and Public Health, University College London, 1-19 Torrington Place, London, UK.

**Address correspondence to:** Eliazar Luna, [eliazar.luna.16@ucl.ac.uk](mailto:eliazar.luna.16@ucl.ac.uk), +44 (0) 020 7679 8252

# Supplementary material

## Supplementary Table 1. Number of deaths by covariate in the analytical sample of the 2003 and 2010 cohort.

| **Variable** | **2003 ENS**  ***n**=3,151** | | **2010 ENS**  ***n**=3,749** | |
| --- | --- | --- | --- | --- |
|  | ***n*** | **%** | ***n*** | **%** |
| **Depressive symptoms** | | | | |
| Yes | 55 | 10.72 | 52 | 8.48 |
| No | 260 | 9.86 | 266 | 8.48 |
| **Sex** | | | | |
| Females | 160 | 9.3 | 173 | 7.77 |
| Males | 155 | 10.84 | 145 | 9.53 |
| **Age groups** |  |  |  |  |
| 18-44 years | 9 | 0.67 | 13 | 0.75 |
| 45-64 years | 47 | 4.59 | 72 | 5.62 |
| 65+ years | 259 | 33.08 | 233 | 32.14 |
| **Marital Status** | | | |  |
| No Partner | 31 | 4.1 | 36 | 4.32 |
| Widowed/Divorced | 135 | 25.52 | 134 | 18.82 |
| With Partner | 149 | 7.98 | 148 | 6.72 |
| **Working Status** | | | | |
| Employed+Student | 32 | 2.29 | 63 | 3.08 |
| Homemaker | 83 | 8.88 | 46 | 5.04 |
| Retired | 153 | 35.33 | 176 | 30.45 |
| Unemployed | 47 | 12.14 | 33 | 15.49 |
| **Years of Education** | | | | |
| 12+ | 8 | 2.06 | 22 | 2.98 |
| 8-12 | 49 | 4.28 | 98 | 4.87 |
| Less than 8 | 258 | 15.95 | 198 | 19.82 |
| **Physical activity** | | | | |
| 3+ times weekly | 8 | 3.05 | 5 | 1.82 |
| 1-2 times weekly | 14 | 4.59 | 2 | 0.71 |
| <4 times monthly | 3 | 2.29 | 6 | 3.49 |
| No sport | 290 | 11.82 | 305 | 10.09 |
| **Smoking** | | | | |
| No | 271 | 13.21 | 260 | 10.86 |
| Yes | 44 | 4 | 58 | 4.28 |
| **High Blood Pressure** | | | | |
| Yes | 190 | 17.54 | 199 | 18.86 |
| No | 125 | 6.04 | 119 | 4.42 |
| **Diabetes** | | | | |
| Yes | 84 | 26.33 | 78 | 21.85 |
| No | 231 | 8.16 | 240 | 7.08 |
| ***Analytical sample size** | | | | |

## Supplementary Figure 1. Kaplan Meier plots of the bivariate association between covariates and mortality by ENS.


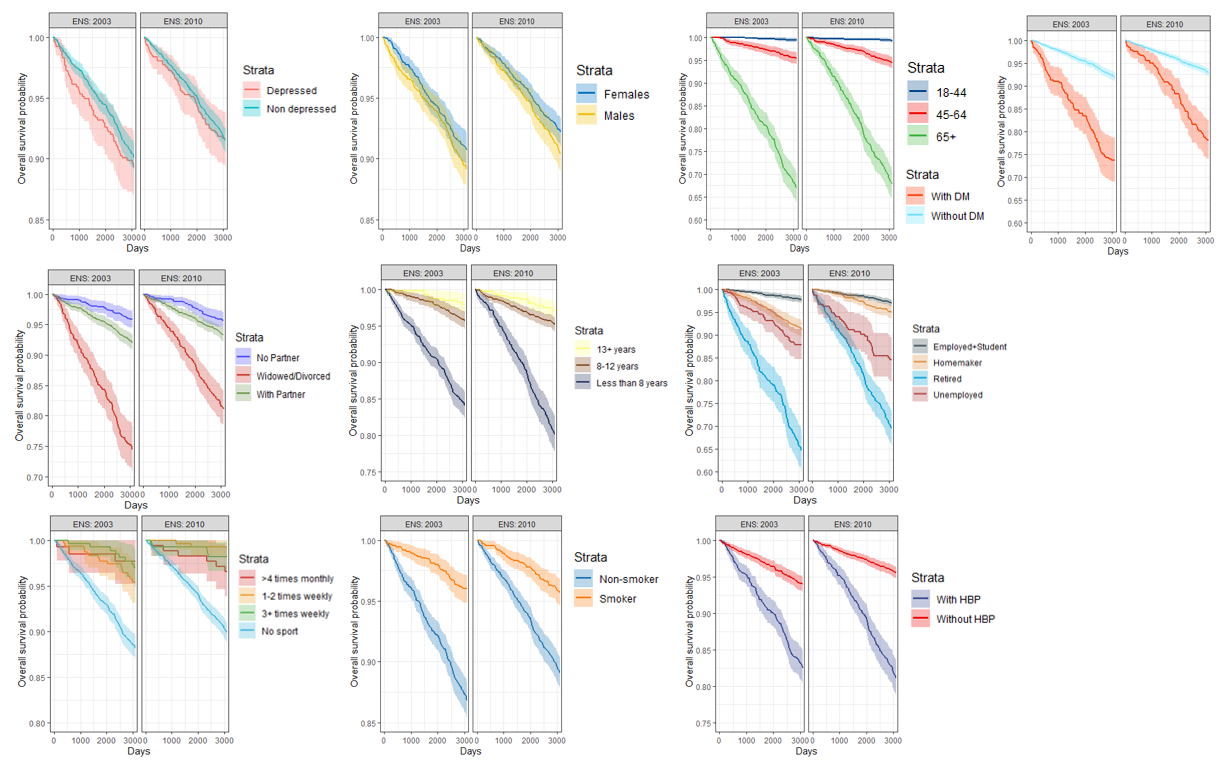


## Supplementary Table 2. Fully adjusted Cox model of the association between depressive symptoms and mortality in the Chilean population using an 8.5-year follow-up.

| **Characteristic** | **2003 Cohort**  ***n*=3,151** | | | **2010 Cohort**  ***n*=3,749** | | |
| --- | --- | --- | --- | --- | --- | --- |
|  | **HR** | **95% CI** | **P-value** | **HR** | **95% CI** | **P-value** |
| **Depressive Symptoms**  **(CIDI-SF≥5)** |  |  |  |  |  |  |
| *Non depressed* | 1 |  |  | 1 |  |  |
| *Depressed* | 1.42 | 1.05, 1.92 | **0.022** | 1.46 | 1.07, 1.99 | **0.017** |
| **Sex** |  |  |  |  |  |  |
| *Females* | 1 |  |  | 1 |  |  |
| *Males* | 1.41 | 1.04, 1.92 | **0.027** | 1.61 | 1.24, 2.08 | **<0.001** |
| **Age groups** |  |  |  |  |  |  |
| *1-year increase* | 1.09 | 1.08, 1.10 | **<0.001** | 1.09 | 1.08, 1.10 | **<0.001** |
| **Marital Status** |  |  |  |  |  |  |
| *No Partner* | 1 |  |  | 1 |  |  |
| *Widowed/Divorced* | 0.80 | 0.53, 1.21 | 0.3 | 0.92 | 0.63, 1.35 | 0.4 |
| *With Partner* | 0.72 | 0.48, 1.07 | 0.11 | 0.86 | 0.59, 1.25 | 0.4 |
| **Years of Education** |  |  |  |  |  |  |
| *12+* | 1 |  |  | 1 |  |  |
| *8-12* | 1.27 | 0.60, 2.73 | 0.5 | 1.10 | 0.69, 1.76 | 0.7 |
| *Less than 8* | 1.52 | 0.74, 3.13 | 0.3 | 1.26 | 0.80, 2.00 | 0.3 |

**Cntd. Supplementary Table 2. Fully adjusted Cox model of the association between depressive symptoms and mortality in the Chilean population using an 8.5-year follow-up.**

| **Characteristic** | **2003 Cohort**  ***n*=3,151** | | | **2010 Cohort**  ***n*=3,749** | | |
| --- | --- | --- | --- | --- | --- | --- |
|  | **HR 95% CI P-value** | | | **HR 95% CI P-value** | | |
| **Working Status** |  |  |  |  |  |  |
| *Employed + Student* | 1 |  |  | 1 |  |  |
| *Homemaker* | 1.62 | 1.01, 2.62 | **0.047** | 1.04 | 0.68, 1.59 | 0.9 |
| *Retired* | 2.19 | 1.43, 3.35 | **<0.001** | 1.36 | 0.96, 1.93 | **0.084** |
| *Unemployed* | 2.09 | 1.30, 3.38 | **0.002** | 3.35 | 2.13, 5.26 | **<0.001** |
| **Physical activity** |  |  |  |  |  |  |
| *3+ times weekly* | 1 |  |  | 1 |  |  |
| *1-2 times weekly* | 1.36 | 0.57, 3.26 | 0.5 | 0.55 | 0.11, 2.85 | 0.5 |
| *>4 times monthly* | 1.09 | 0.29, 4.14 | 0.9 | 2.90 | 0.88, 9.55 | 0.079 |
| *No sport* | 1.65 | 0.82, 3.36 | 0.2 | 2.57 | 1.05, 6.29 | **0.038** |
| **Smoking** |  |  |  |  |  |  |
| *Non-smoker* | 1 |  |  | 1 |  |  |
| *Smoker* | 1.06 | 0.76, 1.49 | 0.7 | 1.26 | 0.92, 1.70 | 0.15 |
| **High Blood Pressure** |  |  |  |  |  |  |
| *Yes* | 1 |  |  | 1 |  |  |
| *No* | 1.05 | 0.83, 1.34 | 0.7 | 0.86 | 0.68, 1.10 | 0.2 |
| **Diabetes** |  |  |  |  |  |  |
| *Yes* | 1 |  |  | 1 |  |  |
| *No* | 0.61 | 0.47, 0.79 | **<0.001** | 0.64 | 0.49, 0.83 | **<0.001** |

## Supplementary Table 3. Sensitivity analyses comparing the assessment between depressive symptoms and mortality adjusted by age and sex, excluding those who died in first 6 months of follow-up by cohort.

| **Variable** | **2003 Cohort**  ***n*=3,355** | | | **2003 Cohort**  **with exclusions**  ***n*= 3,341** | | | **2010 Cohort**  ***n*=** **4425** | | | **2010 Cohort**  **with exclusions**  ***n*=** **4404** | | |
| --- | --- | --- | --- | --- | --- | --- | --- | --- | --- | --- | --- | --- |
|  | **HR** | **95% CI** | **P-value** | **HR** | **95% CI** | **P-value** | **HR** | **95% CI** | **P-value** | **HR** | **95% CI** | **P-value** |
| **Depressive symptoms**  **(CIDI-SF≥5)** |  | | | | | | | | | | | |
| *Non-depressed* | 1 |  |  | 1 |  |  | 1 |  |  | 1 |  |  |
| *Depressed* | 1.53 | 1.15, 2.03 | **0.003** | 1.47 | 1.09, 1.96 | **0.011** | 1.61 | 1.22, 2.12 | **<0.001** | 1.48 | 1.10, 1.98 | **0.009** |
| **Sex** |  | | | | | | | | | | | |
| *Females* | 1 |  |  | 1 |  |  | 1 |  |  | 1 |  |  |
| *Males* | 1.40 | 1.13, 1.73 | **0.002** | 1.33 | 1.07, 1.65 | **0.012** | 1.59 | 1.30, 1.95 | **<0.001** | 1.55 | 1.26, 1.91 | **<0.001** |
| **Age groups** |  | | | | | | | | | | | |
| *1-year increase* | 1.10 | 1.09, 1.11 | **<0.001** | 1.10 | 1.09, 1.11 | **<0.001** | 1.10 | 1.09, 1.11 | **<0.001** | 1.10 | 1.09, 1.11 | **<0.001** |
